# Social, economic and environmental risk factors for acute lower respiratory infections among children under five years of age in Rwanda

**DOI:** 10.1186/s13690-016-0132-1

**Published:** 2016-05-23

**Authors:** Jean-Modeste Harerimana, Leatitia Nyirazinyoye, Dana R. Thomson, Joseph Ntaganira

**Affiliations:** University of Rwanda College of Medicine and Health Sciences, Kigali, Rwanda; Department of Global Health and Social Medicine, Harvard Medical School, Boston, USA

**Keywords:** ALRI, ARI, Demographic and health survey, Community, Integration

## Abstract

**Background:**

In low and middle-income countries, acute lower respiratory illness is responsible for roughly 1 in every 5 child deaths. Rwanda has made major health system improvements including its community health worker systems, and it is one of the few countries in Africa to meet the 2015 Millennium Development Goals, although prevalence of acute lower respiratory infections (4 %) is similar to other countries in sub-Saharan Africa. This study aims to assess social, economic, and environmental factors associated with acute lower respiratory infections among children under five to inform potential further improvements in the health system.

**Methods:**

This is a cross-sectional study using data collected from women interviewed in the 2010 DHS about 8,484 surviving children under five. Based on a literature review, we defined 19 health, social, economic, and environmental potential risk factors, tested bivariate associations with acute lower respiratory infections, and advanced variables significant at the 0.1 confidence level to logistic regression modelling. We used manual backward stepwise regression to arrive at a final model. All analyses were performed in Stata v13 and adjusted for complex sample design.

**Results:**

The following factors were independently associated with acute lower respiratory infections: child’s age, anemia level, and receipt of Vitamin A; household toilet type and residence, and season of interview. In multivariate regression, being in the bottom ten percent of households (OR: 1.27, 95 % CI: 0.85-1.87) or being interviewed during the rainy season (OR: 1.61, 95 % CI: 1.24-2.09) was positively associated with acute lower respiratory infections, while urban residence (OR: 0.58, 95 % CI: 0.38-0.88) and being age 24–59 months versus 0–11 months (OR: 0.53, 95 % CI: 0.40-0.69) was negatively associated with acute lower respiratory infections.

**Conclusion:**

Potential areas for intervention including community campaigns about acute lower respiratory infections symptoms and treatment, and continued poverty reduction through rural electrification and modern stove distribution which may reduce use of dirty cooking fuel, improve living conditions, and reduce barriers to health care.

## Background

Acute respiratory infections are a leading cause of mortality among children under five years of age [1]. In low and middle-income countries, 6.9 million children died in 2011 and about one in five of these deaths was caused by an acute lower respiratory infection (ALRI) [2]. ALRI is characterized by cough accompanied by short, rapid breathing that is chest-related, and is commonly linked to death through co-morbidities with other childhood illnesses [3]. Ninety-seven percent of ALRI cases occur in the developing world with seventy percent of those cases occurring in south Asia and sub-Saharan Africa alone [2].

The most common communicable diseases in Rwanda are malaria, ALRI, HIV and AIDS, diarrhoeal diseases and tuberculosis [4]. In low-resource settings, prevalence of ALRI and other risk factors of child mortality are linked to community and household socioeconomic factors, access to health care, and weather among which housing, nutrition, and indoor air quality stand out [5]. In 2010, four percent of children under-five in Rwanda had symptoms of ALRI in the previous two weeks, which is similar with other countries in sub-Saharan Africa [6].

Interventions such as promotion of health service utilization and integrated management of childhood illnesses in Rwanda have contributed to improved child health outcomes in recent years [7, 8]. Rwanda is one of the few countries in Africa to meet Millennium Development Goal (MDG) 4, the reduction of child mortality; between 2005 and 2010, under-five mortality declined from 152 to 76 deaths per 1,000 live births [6]. Furthermore, close to three-quarters of the population in Rwanda lives within five kilometres of a health facility, 77 % of individuals report not having a geographical barrier to health services, 90 % of children between 12 and 23 months are fully immunised against preventable disease including haemophilus influenza type b, and the rate of access to nationalized health insurance has increased to 78 % [6].

The Rwanda health system is a three-tiered system, with central (teaching hospitals), intermediate (district hospitals), and operational (heath center) levels. An additional level of community health workers help to identify and refer sick individuals to the health system, providing opportunities within the health system to respond to socioeconomic and contextual risk factors as well as medical risk factors for ALRI. In Rwanda, acute lower respiratory infections are managed at health facilities and through community case management.

In spite of health improvements, in 2010 Rwanda was still classified among the countries in the world with high early childhood mortality, 44 % of children under five suffered from chronic malnutrition, and 26 % of the population did not have access to an improved drinking water source [6]. This study identifies social, economic, and weather risk factors for ALRI among children under five to further inform child health programs in communities.

## Methods

**Fig. 1:**
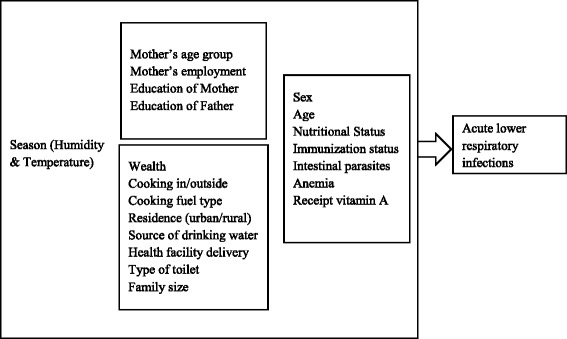
Conceptual framework of risks factors for acute lower respiratory infection

This is a secondary data analysis of the 2010 Rwanda Demographic and Health Survey (RDHS). The 2010 RDHS was a national representative survey of 12,540 households chosen through multi-stage cluster sampling. Of the 13,790 women age 15 to 49 who were eligible to be interviewed, 13,671 (99.1 %) completed an interview. Oral interviews were conducted by trained interviewers at respondents’ homes, and their responses were recorded on paper questionnaires. Women were asked questions about all of their biological children under age five, including questions about acute lower respiratory infections symptoms experienced in the last two weeks. Women reported 8,484 surviving children under age five years. Anthropometry (height, weight) and blood samples were measured on children who stayed in the household on the night before the interview. Therefore, to assess the prevalence of ALRI, mothers were asked if their children under five had been ill with a cough in the two weeks preceding the survey accompanied by short and rapid breathing that was chest related. After excluding six children whose mother’s gave incomplete responses, the analysis included 8,478 children under five. The outcome, suffering from ALRI, was modeled as a binary variable. A literature search in Hinari, Google Scholar, and PubMed was performed to identify child, parent, household, and community factors associated with ALRI, and summarized in a conceptual framework (Fig. 1). Using this, we generated 19 variables for inclusion in this analysis.

Child factors included: age modeled as 0–11 months, 12–23 month, and 24–59 months; sex; underweight defined as being less than –2 standard deviations from the international reference population [9]; receipt of Bacillus Calmette–Guérin (BCG) vaccine; receipt of drugs for intestinal parasites in the last 6 months; anemia status categorized as not anemic (above 10.9 g/dl), mild or moderate anemia (7.0-10.0 g/dl), severe anemia (below 7.0 g/dl), and not-measured; receipt of vitamin A in the last 6 months. Vitamin A is routinely distributed to children age 6–59 months twice-yearly at ‘mothers and child health week’ events which are advertised through campaigns.

Parent factors included: mother’s age group, mother’s employment status, and whether either mother or father had completed secondary school. Household and community factors included whether the household was in the bottom 10 % (calculated from the DHS household wealth factor score); whether the household uses improved cooking fuel (electricity, liquid petroleum gas, natural gas, biogas, kerosene, coal, lignite, charcoal) or unimproved cooking fuel (wood, animal dung, straw/shrubs/grass), whether the household has an improved water source (piped into dwelling, piped to yard/plot, public tap/standpipe, tube well or borehole, protected well, protected spring, bottled water) and whether the household has an improved toilet (flush to piped sewer system, flush to septic tank, flush to pit latrine, ventilated improved pit latrine, pit latrine with slab, composting toilet). The season of interview was defined as rainy (March-May, September-November) and dry (June-August, December-February) season, and residence (urban or rural) was defined in the RDHS.

The analysis was done in two steps; the first step was a bivariate analysis between each covariate and the outcome. We generated percentages and performed chi-square tests comparing children who did and did not suffer from acute lower respiratory infections. Variables associated with ALRI at *p* < 0.1 were kept for multivariable modeling. Two interaction terms were created for bottom 10 % and place of residence, and bottom 10 % and season, and we tested whether these interactions changed the relationship of residence or season with ALRI by more than 10 %. Since there was no evidence of effect modification by low wealth, the interaction terms were dropped from the analysis. None of the covariates were collinear (Pearson’s correlation coefficient *r* > 0.7).

In the second step, manual backward stepwise logistic regression was employed to identify factors that were significantly associated with ALRI at *p* < 0.05. Type of cooking fuel and bottom 10 % were included in all models because they were key risk factors identified in the conceptual framework. Odds ratios and 95 % confidence intervals are presented for the full model and a reduce model of variables remaining after backward stepwise regression. The analysis was performed in Stata version 13 using survey commands to adjust for sampling weights, clustering, and stratification [10].

## Results

The prevalence of ALRI in the two weeks preceding the survey was 4 % (Table 1). The following factors were associated with ALRI at *p* < 0.1 in the bivariate analysis: child’s age, child’s level of anemia, child’s receipt of Vitamin A, toilet type, place of residence, and season of interview. ALRI was particularly high among children suffering from severe anemia (14.6 %), children less than two years (0–11 months: 5.2 %; 12–23 months: 5.1 %), children living in urban areas (5.2 %) and children who did not receive vitamin A in the last six months (4.9 %).

**Table 1:** Bivariate analysis of factors associated with acute lower respiratory infection among children under five in Rwanda, RDHS 2010

Name of Variable	Children in study	Children suffering from ALRI in last two weeks	Chi-squared *p*-value
	Number	Number (Percent)	
CHILD			0.001
Child age		82 (5.2)	
0-11 months	1, 573		
12-23 months	1, 615	82 (5.1)	
24-59 months	5, 411	157 (2.9)	
Child sex			0.104
Boy	4,361	179 (4.1)	
Girl	4,238	144 (3.4)	
Child underweight^a^			0.991
No	3,648	139 (3.8)	
Yes	467	18 (3.8)	
Not measured	4,424	164 (3.7)	
Child received BCG			0.109
No	94	1 (0.9)	
Yes	8,503	323 (3.8)	
Child received intestinal drugs in last 6 months			0.119
No	94	4 (4.4)	
Yes	8,503	306 (3.6)	
Anemia level^a^			0.083
Not anemic	2,316	74 (3.2)	
Mild or moderate	1,441	60 (4.2)	
Severe	17	2 (14.6)	
Not measured	4,424	164 (3.7)	
Child received vitamin A in last 6 months			0.040
No	1,109	54 (4.9)	
Yes	7,484	269 (3.6)	
Child delivered at a health facility			0.326
No	2,625	89 (3.4)	
Yes	5,969	233 (3.9)	
PARENT			
Mother current age			0.178
<21 years	273	14 (5.3)	
21+ years	8,326	308 (3.7)	
Mother employment status			0.225
Not working or self-employed agriculture	7,488	269 (3.6)	
Working	1,100	50 (4.6)	
Mother education level			0.210
Less than secondary	7,837	282 (3.6)	
Secondary or high	762	37 (4.9)	
Partner education level^a^			0.406
Less than secondary	7,155	257 (3.6)	
Secondary or higher	882	40 (4.4)	
HOUSEHOLD			0.109
Bottom 10 % (wealth score)			
No	7,737	278 (3.6)	
Yes	862	42 (4.9)	
Cooking fuel type^a^			0.417
Unimproved	7,761	279 (3.6)	
Improved	743	33 (4.5)	
Residence			0.064
Rural	7,566	272 (3.6)	
Urban	1,033	54 (5.2)	
Household size			0.195
>5 members	3,796	152 (4.0)	
<=5 members	4,803	168 (3.5)	
Source of drinking water^a^			0.322
Unimproved	2,423	99 (4.1)	
Improved^b^	6,088	213 (3.5)	
Improved toilet^a^			0.058
Unimproved	2,308	101 (4.4)	
Improved^c^	6,200	217 (3.5)	
Season			0.001
Dry	4,837	144 (3.0)	
Rainy	3,762	177 (4.7)	

^a^Missing observations

^b^Includes: piped water, piped into dwelling, piped to yard/plot, public tap/standpipe, tube well or borehole, protected well, protected spring, bottled water

^c^Includes: ventilated improved pit latrine, pit latrine with slab

After adjusting for other covariates in the multivariate regression (Table 2), children belonging to households in the bottom 10 % had 1.27 times the odds (95 % CI: 0.85-1.87) of ALRI than children living in better off households, and children of mothers interviewed during the rainy season had 1.61 the odds of ALRI (95 % CI: 1.24-2.09) than the dry season. The following factors were protective against ALRI: living in urban versus rural areas (OR: 0.58, 95 % CI: 0.38-0.88), and being age 24–59 months compare to 0–11 months (OR: 0.53, 95 % CI: 0.40-0.69).

**Table 2:** Multivariate logistic regression model of acute lower respiratory infection among children under five in Rwanda, RDHS 2010

Variable	AOR (95 % CI)	AOR (95 % CI)
	Full model	Reduced Model
Cooking fuel type		
Not improved	ref	ref
Improved	0.89 (0.51-1.54)	0.93 (0.55-1.58)
Bottom 10 % (wealth score)		
Not bottom 10 %	ref	ref
Bottom 10 %	1.01 (0.65-1.58)	1.27 (0.85-1.87)
Child age		
0-11 months	ref	ref
12-23 months	0.96 (0.65-1.42)	0.93 (0.67-1.31)
24-59 months	0.54 (0.39-0.77)	0.53 (0.40-0.69)
Anemia level		
Not anemic	ref	
Mild or moderate	1.11 (0.78-1.57)	
Severe	3.49 (0.68-17.90)	
Not measured	1.06 (0.79-1.43)	
Child received vitamin A in last 6 months		
No	ref	
Yes	1.03 (0.69-1.52)	
Residence place		
Rural	ref	ref
Urban	0.50 (0.33-0.75-2.87)	0.58 (0.38-0.88)
Improved toilet		
Unimproved	ref	
Improved	0.82 (0.62-1.12)	
Dry rainy season		
Dry season	ref	ref
Rainy season	1.59 (1.20-2.09)	1.61 (1.24-2.09)

*CI* confidence interval, *AOR* adjusted odds ratio, *ref* reference

## Discussion

This analysis identified several socioeconomic and weather risk factors for acute lower respiratory infections in Rwanda. The findings revealed that rainy season is associated with acute lower respiratory infections which is consistent with studies in other similar countries [11]. There are both biological and social reasons that acute lower respiratory pathogens differ across geographic and climate zones. Studies from temperate and tropical regions found that temperature and humidity can explain influenza seasonality by diminishing the host immunity through, for example, the inhalation of cold air which causes vasoconstriction and reduction of blood flow while dry conditions, on the other hand, can reduce mucociliary clearance [12, 13]. Social reasons that link ALRI and geography are that many households in tropical regions face crowding due to low income; crowding becomes more problematic during heavy rain which forces people to stay inside and can increase exposure to ALRI [5]. Low income can have a similar effect on ALRI in colder regions where winter conditions cause people to stay inside with poor ventilation [14, 15].

Given the greater environmental and social risks for acute lower respiratory infections during the rainy season, campaigns via public radio, television, and community dramatization should describe these risks to the public, and encourage households to ventilate homes and seek treatment for respiratory illness. The Rwanda Environment Management Authority has been producing drama-film sketches on Rwanda broadcast television for years tackling different aspects of sustainable development and environment protection [16]. Use of film sketches like these to raise community awareness about respiratory diseases before and during the rainy season would be an example of creative solutions that leverage existing resources.

This study found that children living in poor economic conditions are more likely to suffer from acute lower respiratory infections than children living in better off households. This finding is common around the world [17]. Globally, poor living conditions present risk factors for illness and are associated with inadequate utilization of primary health care [18]. In Rwanda, the Government has tried to overcome typical barriers to health care among the poor through a national low-cost health insurance scheme which is very popular, and community-based case management of childhood illness targetting children under five [19], still children from poor households face a problem of healthcare utilization [20]. Thus, broad rural poverty reduction and social protection strategies are likely needed still. The Second Economic Development and Poverty Reduction Strategy for 2013–2018 aims to implement Rwanda’s ambitious Vision 2020 plan, to move the nation from low-income to middle-income status [21]. The strategy includes reducing poverty to below 30 % through national electrification programs and shifting cooking practices to modern biomass fuels, restructuring the economy to focus on services and industry, and implementing strong environmental protection and restoration inititives that could create jobs in rural areas.

The following factors were protective against acute lower respiratory infections. First, children between 24–59 months of age had a lower risk of ALRI than children age 0–11 months which is likely because children build their immunity systems over time enabling them to fight off infectious agents from the environment [22]. Second, urban residence was also protective against acute lower respiratory infections. Rwanda’s urban population is expected to increase substantially from 17 % in 2012 to 30 % in 2032 [4]. While Rwanda’s government closely monitors air pollution, and prioritizes modern transportation, renewable energy and environmental protection in its development strategies, it is wise for future studies to monitor links between urbanity, air pollution, and ALRI in Rwanda as urban air pollution is a key risk factor for ALRI in other similar settings [23]. Type of cooking fuel was not associated with ALRI in either the bivariate or multivariate analysis. This is contrary to a number of other studies which show a strong association between ALRI and unimproved cooking fuel [24–26] due to poor ventilation [27, 28]. The assumption of this null result was a statistical artefact because 98 % of household used unimproved cooking fuel and there was lack of statistical variability. The fact that the vast majority of households still cook with unimproved fuel, which is a major known risk factor for acute lower respiratory infections, should be addressed. Lack of wealth, particularly access to electricity, is a primary barrier to using improved cook stoves [29].

Two key limitations of this analysis were that we were not able to measure causal effects because the analyses was based on cross-sectional data, and the dataset did not include some socioeconomic variables including duration of cooking, or use of multiple fuels together such as wood and charcoal.

## Conclusion

The study found that environmental and socioeconomic factors were associated with childhood acute lower respiratory infections, and these results point toward potential areas for intervention including community campaigns about acute lower respiratory infections symptoms and treatment of respiratory infection. Rwanda has initiated many strategies to reduce poverty including rural electrification and modern stove distribution which may reduce use of dirty cooking fuel, improve living conditions, and reduce barriers to health care in the future.

## Abbreviations

ALRI: acute lower respiratory infection
AOR: adjusted odds ratio
CI: confidence interval
RDHS: Rwanda demographic and health survey
